# Diarrhoea Management using Over-the-counter Nutraceuticals in Daily practice (DIAMOND): a feasibility RCT on alternative therapy to reduce antibiotic use

**DOI:** 10.1186/s40814-021-00850-y

**Published:** 2021-06-15

**Authors:** Yanhong Jessika Hu, Xudong Zhou, Shanjuan Wang, Merlin Willcox, Colin Garner, David Brown, Taeko Becque, Beth Stuart, Zongru Han, Qin Chang, Michael Moore, Paul Little

**Affiliations:** 1 Murdoch Children’s Research Institute, Royal Children’s Hospital, Parkville, Victoria Australia; 2 Department of Paediatrics, The University of Melbourne, Parkville, Victoria Australia; 3 School of Public Health, Zhejiang University, 866 Yuhangtang Road, Zhejiang, 310058 Hangzhou China; 4 Affiliated Hospital of Jiading District Centre, Shanghai Institute of Health Science, No. 1 Chengbei Rd, Jiading Qu, Shanghai Shi, 201800 China; 5 Primary Care Research Centre, University of Southampton, Aldermoor Health Centre, Aldermoor Close, Southampton, SO16 5ST United Kingdom; 6 Antibiotic Research UK, Genesis 5, York Science Park, Heslington, York, YO10 5DQ United Kingdom; 7 Alchemy Biomedical Consulting, St Johns Innovation Centre, Cowley Road, Cambridge, CB4 0WS, United Kingdom

**Keywords:** Diarrhoea, Antibiotic management, Over-the-counter, Nutraceuticals, Feasibility RCT, Alternative therapy, Turmeric (curcumin-active ingredient)

## Abstract

**Background:**

Although rarely indicated, antibiotics are commonly used for acute diarrhoea in China. We conducted a randomised, double blind exploratory clinical trial of loperamide, berberine and turmeric for treatment of acute diarrhoea.

**Methods:**

Adults with acute uncomplicated diarrhoea aged 18 to 70 were randomised to 4 groups: (A) loperamide; (B) loperamide and berberine; (C) loperamide and turmeric; (D) loperamide, berberine and turmeric. All participants were given rescue ciprofloxacin for use after 48 h if symptoms worsened or were unimproved. Primary endpoints were feasibility and ciprofloxacin use during the 2-week follow-up period. Semi-structured interviews were conducted following recruitment and were analysed thematically. Recruiting doctors, delivery pharmacists and research assistants were blinded to treatment allocation.

**Results:**

Only 21.5% (278/1295) of patients screened were deemed eligible, and 49% (136/278) of these consented and were entered into the final analysis. Most participants had mild symptoms, because most patients with moderate or severe symptoms wanted to be given antibiotics. Follow-up was good (94% at 2 weeks). Only three participants used rescue antibiotics compared to 67% of acute diarrhoea patients in the hospital during the recruitment period. The median symptom duration was 14 h in group B (interquartile range (IQR) 10-22), 16 h in group D (IQR 10-22), 18 h in group A (IQR 10-33) and 20 h in group C (IQR 16-54). Re-consultation rates were low. There were no serious treatment-related adverse events. Most interviewed participants said that although they had believed antibiotics to be effective for diarrhoea, they were surprised by their quick recovery without antibiotics in this trial.

**Conclusion:**

Although recruitment was challenging because of widespread expectations for antibiotics, patients with mild diarrhoea accepted trying an alternative. The three nutraceuticals therapy require further evaluation in a fully powered, randomised controlled trial among a broader sample.

**Trial registration:**

ChiCTR-IPR-17014107

**Supplementary Information:**

The online version contains supplementary material available at 10.1186/s40814-021-00850-y.

## Highlights

### What is already known about this subject

- Unnecessary antibiotic use for acute diarrhoea is very common in primary care in China
- Patients and doctors in China expect treatment for symptom relief
- There are safe nutraceuticals already available for acute diarrhoea treatment
- No study has evaluated if it is feasible to replace antibiotics with nutraceuticals for acute diarrhoea

### What are the new findings

- Patients and doctors found it acceptable to use alternatives to antibiotics but only for cases of mild diarrhoea
- Loperamide, berberine and turmeric as alternative therapies allowed doctors to forego antibiotics in China for patients with mild diarrhoea, but require further evaluation in a fully powered randomised controlled trial
- Conducting studies on reducing unnecessary antibiotic use in China is challenging

### How might it impact on clinical practice in the foreseeable future

- Provide a therapeutic alternative to antibiotics for acute diarrhoea management
- After an adequately powered study, if shown to be effective, nutraceuticals could become standard therapy for managing acute diarrhoea

## Introduction

Acute diarrheal disease is still a major global burden [1–3]. Despite the limited benefit and potential harm from antibiotic use, acute diarrhoea is commonly treated with antibiotics in low- and middle-income countries (LMICs) [4–6]. Recent audits demonstrate that antibiotics are currently the standard treatment in many places in China [5]. There is a clear relationship between excessive antibiotic prescribing and antibiotic resistance [7, 8], which is a major threat to public health as new antibiotic drug development takes a long time and requires very significant investment [9]. China is one of the highest antibiotic consuming countries, with high rates of antibiotic resistance reported [10].

An effective non-antibiotic treatment for acute diarrhoea could relieve the pressure on Chinese doctors to prescribe antibiotics. Patients in primary care are often frustrated with the lack of effective interventions for self-limiting illnesses and a majority (72%) report that they expect to receive a prescription for something to help their symptoms [11], especially when they are poor and have spent precious resources to travel to see a doctor. Telling them that their problem will resolve spontaneously may lead to conflicts [12]. There is thus a significant incentive for Chinese doctors to prescribe something to meet patients’ expectations.

### Non-antibiotic treatments for diarrhoea

Berberine is a natural product contained in many herbal medicines which are traditionally used for the treatment of diarrhoea [13]. It is likely to be effective in acute diarrhoea [14, 15], probably working through both antibacterial and anti-inflammatory effects, protecting against lipopolysaccharide induced intestinal injury by binding to TLR4/MD-2 receptors [16, 17]. Berberine use has been associated with changes in gut microbiota and an antidiarrheal effect [18]. Doses up to 400 mg daily were found to be effective in two trials in acute diarrhoea [14, 15, 19].

Curcumin (from turmeric) affects a range of cell modulating pathways and has antibacterial, antiviral, antioxidant and anti-inflammatory effects [20–22]. It is likely to be effective in diarrhoea of either infectious or non-infectious origin [21, 23–25]. One study of curcumin showed a rapid resolution of HIV-related diarrhoea [25] at a dose of 500 mg three times daily and doses up to 12000 mg per day have been used safely [23].

Loperamide not only is a widely used anti-motility agent but also acts synergistically with antibacterial agents [26]. A combination of berberine, turmeric and loperamide addresses a wide range of mechanistic pathways which are potential causes of acute diarrhoea.

### Preliminary data and experience

Anecdotal experience with the combination of turmeric, berberine and loperamide for traveller’s diarrhoea has yielded dramatic results in 6 participants (DB, personal communication), faster than would be expected by loperamide alone [27]. All these agents are widely available over the counter, so this combination could provide an accessible treatment for acute diarrhoea globally and reduce inappropriate antibiotic use. However, although it is plausible that all three components are effective, it has not been clearly shown whether all three components are really needed for effective treatment. Before a fully powered trial can be justified, a smaller exploratory trial to document the feasibility and acceptability of using alternatives to antibiotics is warranted.

### Objectives of this study

*The primary feasibility objectives of this trial were:*

- To assess feasibility of recruitment
- To assess retention and follow-up of recruited participants
- To assess completion and return of symptom diaries
- To identify barriers and facilitators to implement of this trial

*The secondary patient-centred objectives were the following:*

- To estimate antibiotic use in intervention groups compared to usual care
- To estimate the effect of combinations of nutraceuticals plus loperamide on duration and severity of acute diarrhoea
- To estimate the incidence of side-effects in the intervention groups

## Methods and analysis

The CONSORT extension statement checklist for pilot studies [28] was used as a guide to ensure complete and transparent reporting of our study (see Additional file 1).

### Recruitment

Recruitment took place from 10th January to 30th September 2019, in a tertiary care hospital outpatient setting in China, where up to 75% of patients with acute diarrhoea are given antibiotics [4].

Outpatient clinical doctors screened participants’ eligibility during usual clinical consultations, then a researcher assistant introduced the trial to eligible participants, invited them to take part and obtained their consent. We included adults aged 18 to 70 presenting with acute diarrhoea, defined as at least 3 unformed stools in the previous 24 h, and with a duration of less than 7 days, without complications [29]. We excluded participants with vomiting as the most prominent symptom, visible blood in the stool, temperature greater than 39 degrees, suspected to have acute cholera or pseudomembranous colitis, who were immunocompromised, who had allergy to any of the proposed agents, symptom duration more than 7 days, pregnant women, patients with known chronic bowel disease, established ischaemic heart disease or a history of cardiac arrhythmias, and prolonged QT interval.

### Randomisation

All recruiting doctors had packages, each of which had a unique computer-generated random study number together with a coded intervention group number prepared by the study statistician (BS), who was not involved in the implementation of group allocation. This package included a patient enrolment screening chart, consent information form, diary booklet, stool test form and medicine number. Each form was affixed with the study number. All these were prepared before recruitment was initiated. After recruitment, the participant went to the pharmacist who dispensed the corresponding medication pack. Onsite research assistants (RAs) were available to further answer questions for participants. Recruiting doctors, delivery pharmacists and research assistants were blinded to treatment allocation.

### Intervention

Each participant pack contained a 3-day supply of one of the following combinations, and each combination was to be used after each loose stool up to 3 times per day to continue until the diarrhoea stopped:

- Group A, loperamide 4 mg initially then 2 mg following each loose stool, up to three times per day.
- Group B, loperamide used as in group A and berberine100 mg up to four times daily following each loose stool.
- Group C, loperamide used as in group A and turmeric 500 mg up to three times daily following each loose stool.
- Group D, the combination of all 3 (loperamide, berberine and turmeric all as above).

A ‘rescue’ antibiotic (ciprofloxacin 750 mg stat) was given to be used only if symptoms were not starting to settle within 24 h, after telephone or face-to-face assessment by the designated doctor.

We included non-active nutraceuticals in the medication packs (amino acids) [30] for those individuals not receiving triple therapy so that every individual had three medication containers to use.

### Materials

Loperamide was obtained from onsite hospital which was produced from Xi’an Janssen Pharmaceutical Co. Ltd. as 2 mg tablets. Berberine was obtained from onsite hsopital and it was from ReYoung Pharmaceutical Co. Ltd. as 100 mg tablets. Turmeric containing 6-8% curcumin was obtained from Nu U Nutrition, York, UK, as 500 mg capsules. Ciprofloxacin 750 mg was obtained from onsite hospital and it was from Shangdong Qilu Pharmaceutical Co. Ltd. Amino acid control tablets were obtained from Zhejiang CONBA Pharmaceutical Co. Ltd. CONBA G20120506 as 0.8 g/tablets.

### Outcome measures

#### Primary feasibility outcomes

Feasibility outcomes included recruitment rate, exclusion rate and reasons, rejection rate and reasons, completion rate of the diary, compliance rate with the trial medications, withdrawal rate and follow-up rate. Patients were counted as lost to follow-up after three attempts at different time at each follow-up time. Each participant was asked to complete a daily symptom diary and compliance with the trial medications which has been used successfully in trials of medicines for diarrhoea [27]. The diary book recorded symptoms and medicines (medicines from trial and other resources) taken for 7 days starting from recruitment day 1 and asked to return at day 7. All participants were instructed to seek medical assistance again in the event that symptoms progressed. Follow-up was scheduled at 24 h (telephone, clinic visit if needed), 48 h (clinic visit), day 7 (clinic visit) and day 14 (telephone).

#### Secondary patient-centred outcomes

Exploratory outcomes included use of rescue antibiotics in 24 h, the duration of symptoms, the proportion with diarrhoea resolved at 24 h, and the severity of symptoms.

#### Use of antibiotics

We asked participants about antibiotic use including the “rescue” ciprofloxacin and any antibiotics from other sources through follow-up with doctors and phone calls from research assistants. This information was also recorded in the patient diary.

#### Severity of symptoms

The severity score was documented in the first 48 h because this is when symptoms are the most severe and nutraceuticals might make an important difference. The diary book recorded the number of stools during the previous 24 h for 7 days, the consistency of the last stool and the time since the last loose stool. The diary also recorded the severity of symptoms: diarrhoea, vomiting, nausea, abdominal pain, anal burning, fever, disturbed sleep, feeling generally unwell and interference with normal activities. Each symptom was scored on a Likert scale which has been shown to be valid and sensitive to change for a variety of infections, with up to 80% predictive sensitivity and more than 70% predictive specificity [31–34]. Scores ranged from 0 to 6: 0=no problem, to 6=as bad as it could be. Also documented was the duration of symptoms rated at least moderately bad (3) and the time taken for all symptoms to be rated as very little or no problem (1 or 0). Scores were grouped as 0-2 for mild, 3-4 for moderate and 5-6 for severe. A detailed severity and reporting process is provided in Additional file 2.

#### Incidence of side-effects

In the diary booklet, we asked participants to write down the potential side effects as below.

*Do you think you may have had potential side effects of medication?*

*If yes, please specify ______________________________*

Also, this question was listed in the follow-up with doctors and phone calls from research assistants. Potential side effects included constipation, skin rash and nausea.

### Statistical analysis

#### Sample size estimation

In addition to feasibility outcomes, we aimed to recruit a sufficient sample to detect a difference between 50% using rescue antibiotics in the loperamide group and 15% in any other intervention group (for alpha 0.05 and 80% power), since 50% are likely to have resolved in 24 h [35, 36]. Assuming the median time for resolution in the loperamide group is 12 h (at the lower end of prior trial estimates [37]), we estimated that a sample of 30 per group with 1:1:1:1 ratio, which would allow us to detect a reduction in duration of loose stool to 8 h, or a hazard ratio of 2.1 with a slight over-enrolment to account for loss to follow-up.

All analyses were conducted following the intention-to-treat principle. All recruited cases were included, and there was no imputation of missing data. Descriptive statistics were used to report feasibility and clinical outcomes. Continuous variables were summarised as mean (sd) if normally distributed or median (range) for skewed data. Categorical data were summarised as counts and percentages. Descriptive statistics were used to describe side effects as total number, proportion and mean. If possible, multivariable regression analyses adjusting for number of loose stools at baseline, mean severity and prior duration were conducted for exploratory outcomes. Logistic regression was used for binary outcomes (antibiotic use and proportion with diarrhoea resolved at 24 h), linear regression was used for mean symptom severity, and Cox regression was used for duration until diarrhoea resolved, where possible. All analyses were performed using STATA 16 (Stata, College Station, TX, USA).

#### In-depth interviews

We conducted semi-structured qualitative interviews of patients and interviewed recruiting doctors and on-site research assistants after trial recruitment was completed. We planned to interview both patients who had declined to participate and those who had participated in the trial. We selected participants by study subject number and adjusted to ensure each group had a similar number of participants. Participants were invited to provide verbal consent. Interviews were conducted during the last week of recruitment by YJH. Face-to-face interviews were done on site. For patients who had completed follow-up and remained at home, we conducted telephone interviews. Interviews were discontinued once we reached data saturation. We aimed to understand which trial procedures did and did not work well, whether they were willing to recommend this therapy and suggestions for a future scale up study. Patients and doctors were also asked about what treatment they normally use for acute diarrhoea and whether they would be willing to recommend the trial therapy to others.

## Results

### Primary feasibility outcomes

#### Recruitment

**Fig. 1:**
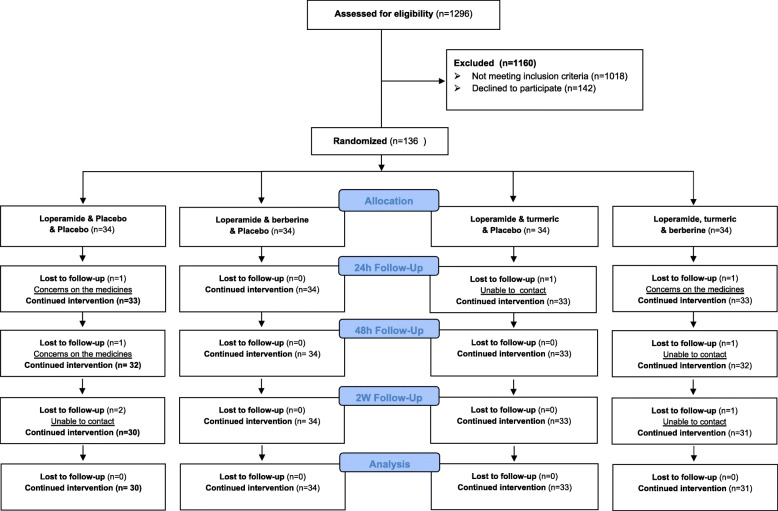
Consort report of patients’ pathway and the reasons for declined patients

In total, 1296 patients were screened, 1160 were excluded and 136 were recruited. The majority of exclusions were either the doctor’s assessment that the patient was too severely unwell (57%) or the patients were already on antibiotics (9%) (Fig. 1).

**Fig. 2:**
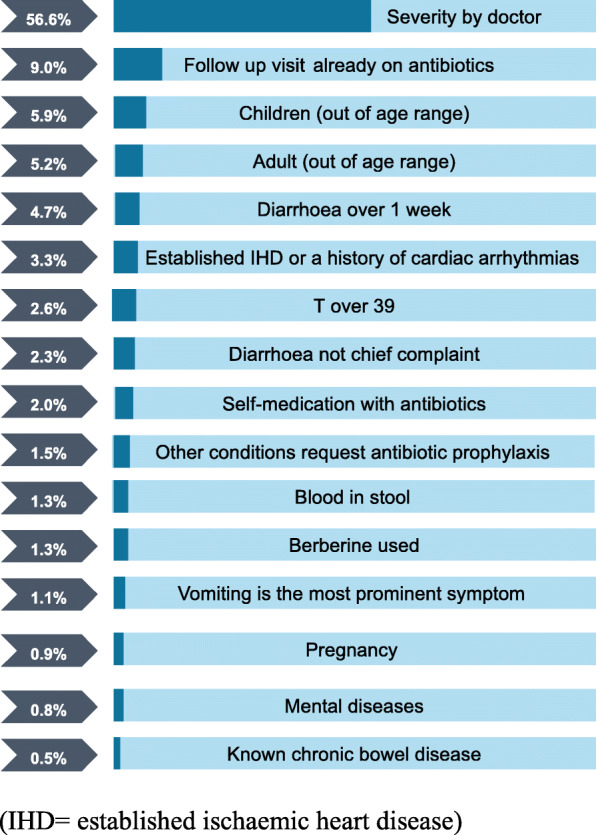
Proportion of excluded patients by reason

Among the 278 eligible patients, 142 (51%) refused to participate (Fig. 2); 60 gave no reason (42%), 36 (25%) did not have time, 19 (13%) did not trust the trial medicines and 15 insisted on receiving an injection (11%). 13 were advised by family member not to join or insisted use antibiotics (9%).

#### Diary completion

The overall diary completion and return rate was 92% (125/136) with no significant differences between the groups. The overall trial medicine compliance rates in total were 100% at day 1, 94% at day 2; 94% at day 3 and with similar rates to diary completion rates among 4 groups at day 7.

#### Baseline characteristics of recruited patients

Baseline characteristics are presented in Table 1. Overall, gender was fairly balanced across randomised groups. Most patients were young or middle-aged (mean 33 years, SD 12 years), and 39% were originally from Shanghai, whilst the rest had migrated there for work. The median number of loose stools in the last 24 h before treatment was 4 (IQR 4-5.5). Symptom severity was mild with an overall mean of 1.1 (sd 0.7) and was balanced across randomised groups. The response rate for baseline diarrhoea severity was high among 136 recruited participants, 125 (92%) recorded diarrhoea severity. Response rates for other baseline symptom items were lower: 69 (51%) reported abdominal cramping severity, 29 (21%) reported nausea severity and few patients (<10%) reported the remaining symptom items (vomiting, generally unwell, fever, muscle ache, headache, disturbed sleep, interference with normal activities, interference with social activities).

**Table 1:** Baseline characteristics

	Number	Group A Loperamide only (*N*=34)	Group B Loperamide and berberine (*N*=34)	Group C Loperamide and turmeric (*N*=34)	Group D Loperamide, turmeric and berberine (*N*=34)
Gender—male (n, %)	136	18 (52.9)	20 (58.8)	17 (50.0)	22 (64.7)
Age (mean, sd)	134	33.0 (10.2)	29.9 (10.7)	34.7 (12.3)	34.0 (13.1)
Hometown—from Shanghai	136	13 (38.2)	11 (32.4)	16 (47.1)	13 (38.2)
Number loose stools in last 24 h (median, IQR)	134	4.5 (4, 5)	4 (3.5, 5.5)	4 (3, 5)	4 (3.5, 6)
Mean symptom severity^a^ (mean, sd)	128	0.9 (0.5)	1.3 (0.8)	1.1 (0.7)	1.1 (0.7)
Prior duration of loose stools (median, IQR)	127	19 (8, 35)	13.5 (9, 28)	13 (9, 33)	18 (11, 30)

^a^Symptoms (nausea, vomiting, abdominal cramping, generally unwell, fever, muscle ache, headache, disturbed sleep, interference with normal activities, interference with social activities, diarrhoea) rated on a scale from 0 to 6, where 0 indicates no problem, 1 indicates ‘very little problem’, 2 indicates ‘slight problem’, 3 indicates ‘moderately bad’, 4 indicates ‘bad’, 5 indicates ‘very bad’ and 6 indicates ‘as bad as it could be’

#### Follow-up

**Fig. 3:**
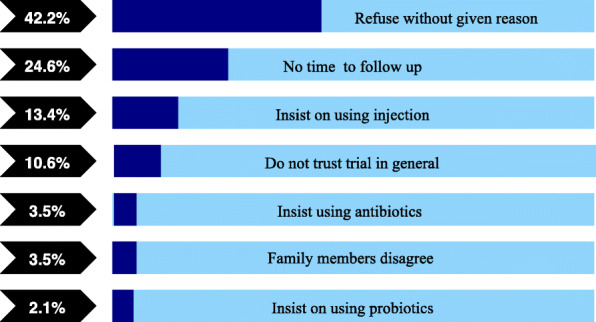
Reasons for refusal to participate in the trial

Follow-up rates were high, with 133 people (98%) followed up at 24 h, 131 (96%) at 48 h, 128 (94%) at 1 week, and 128 (94%) at 2 weeks. In total, there were 8 patients who withdrew or were lost to follow-up. Among those withdrawing, three were concerned with side effects from the trial medicines and 5 could not be reached after 24 h or 48 h (Fig. 3).

### Secondary patient-centred outcomes

#### Antibiotic use

Overall, only 3 participants took antibiotics during the study period. No patients in groups B (berberine + loperamide), C (turmeric + loperamide) or D (berberine and turmeric + loperamide) were advised by a recruitment doctor to take rescue antibiotics. Only 2 patients in group A (loperamide only) were advised to take rescue antibiotics. One person took rescue antibiotics in group C (loperamide and turmeric) without being advised to do so.

#### Effect of diarrhoea treatment on antibiotic use, duration and severity of symptoms

**Fig. 4:**
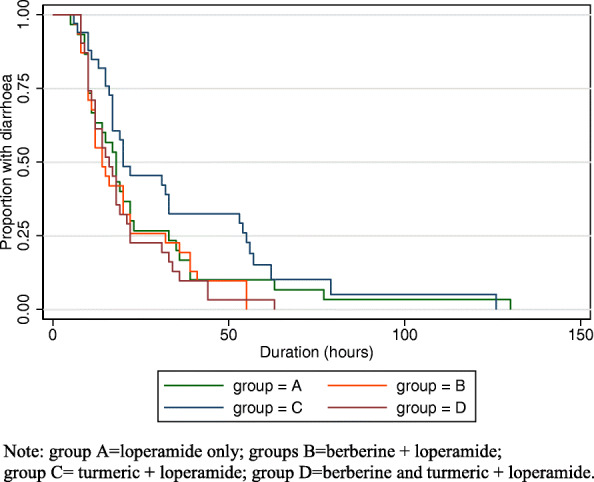
Kaplan-Meier curves for duration of diarrhoea

Crude outcomes along with adjusted estimates for antibiotic use, duration of diarrhoea, proportion of patients with diarrhoea resolved at 24 h and mean symptom severity during the first 48 h are presented in Table 2. There was no statistically significant difference in duration of diarrhoea between groups, nor in the proportion of patients reporting diarrhoea resolved at 24 h. Kaplan Meier curves for the duration of diarrhoea are presented in Fig. 4. The curves for group C (turmeric + loperamide) and D (berberine and turmeric + loperamide) cross the curve for group A (loperamide only), indicating that the proportional hazards assumption does not hold, so hazard ratios have not been calculated.

**Table 2:** Effectiveness of diarrhoea treatments on antibiotic use, duration and severity of symptoms

	Number analysed	Group A Loperamide only	Group B Loperamide and berberine		Group C Loperamide and turmeric		Group D Loperamide and turmeric and berberine	
		Crude outcome	Crude outcome	Adjusted^a^ estimate (95% CI)	Crude outcome	Adjusted^a^ estimate (95% CI)	Crude outcome	Adjusted^a^ estimate (95% CI)
Antibiotic use (n, %)	133	2 (6.0)	0 (0.0)	-	0 (0.0)	-	0 (0.0)	-
Duration until diarrhoea resolved in hours (median, IQR)	127	18 (10, 33)	14 (10, 22)	-	20 (16, 54)	-	16 (10, 22)	-
Proportion diarrhoea resolved^b^ at 24 h (n, %)	132	22 (64.7)	25 (73.5)	OR 1.5 (0.5, 4.3)	19 (55.9)	OR 0.7 (0.3, 1.8)	24 (70.6)	OR 1.3 (0.5, 3.6)
Mean symptom severity^c^ during first 48h (mean, SD)	125	0.5 (0.6)	0.4 (0.4)	MD −0.1 (−0.4, 0.2)	0.6 (0.7)	MD 0.1 (−0.2, 0.4)	0.5 (0.5)	MD 0.0 (−0.3, 0.3)

^a^Adjusted for number of loose stools at baseline, mean severity at baseline and prior duration

^b^Logistic regression of proportion diarrhoea resolved on group, adjusted for variables listed above^a^. Estimate reported as odds ratio (OR, 95% CI) compared to reference group A

^c^Linear regression of mean symptom severity on group, adjusted for variables listed above*. Estimate reported as mean difference (MD, 95% CI) compared to reference group A

#### Side-effects and re-consultations (Table 3)

No constipation was reported for groups B or C at 24 or 48 h. Constipation affected a small number of patients in groups A and D, and the mean severity of constipation at 24 and 48 h was mild. No rashes were reported at 24 or 48 h. There were very small numbers of unscheduled re-consultations and re-contacts.

**Table 3:** Side effects and re-consultation

	Number analysed	Group A Loperamide alone	Group B Loperamide and berberine	Group C Loperamide and turmeric	Group D Loperamide and turmeric and berberine
Mean severity of constipation^a^ at 24 h (mean, sd)	12	1.3 (0.6)	-	-	0.8 (0.4)
Mean severity of constipation at 48 h (mean, sd)	6	1.0 (1.0)	-	-	1.0 (1.4)
Re-consultation (n, %)	125	1 (3.6)	1 (2.9)	0 (0.0)	1 (3.3)
Re-contact (n, %)	125	1 (3.6)	1 (2.9)	1 (3.0)	(0.0)

^a^Constipation severity rated on a scale from 0 to 6, where 0 indicates no problem, 1 indicates ‘very little problem’, 2 indicates ‘slight problem’, 3 indicates ‘moderately bad’, 4 indicates ‘bad’, 5 indicates ‘very bad’ and 6 indicates ‘as bad as it could be’

#### Usual care for acute diarrhea

We extracted data from the hospital information system for all patients diagnosed with only acute diarrhoea without any comorbidities over the same time period. In total, 1367 patients in the same age range were analysed. There were 757 male (55%), and 610 female (45%) patients. Mean age was 41 years old. The antibiotic use rate in usual care overall during the study period was 67% (914/1367) whilst a rate of 2% (3/136) was observed in this trial. Further analysis showed that of patients receiving antibiotics in usual care, 64% (581/914) received these by injection. Of those given antibiotics, the majority (60%) received one antibiotic whilst 38% received two antibiotics and about 1.5% received more than 2 antibiotics. Sixty-three percent of them were given levofloxacin and 32% a 3rd generation cephalosporin.

#### Interview study

**Fig. 5:**
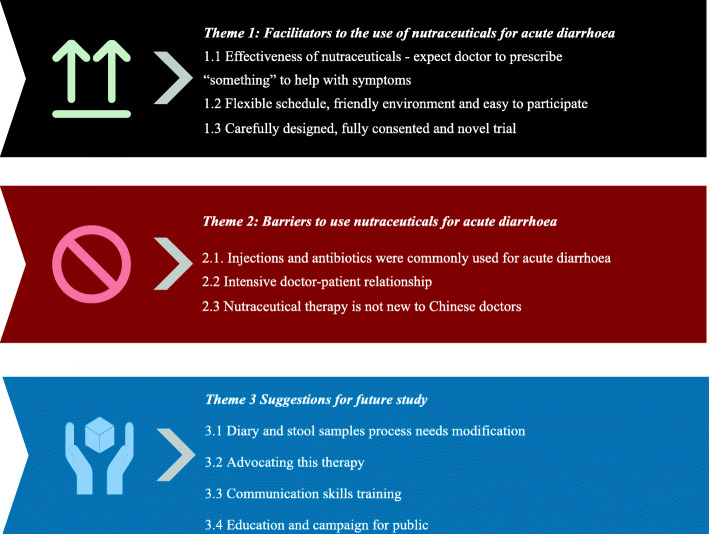
Three themes from interviews

Of 30 participants approached, seven participants did not answer, six interviews could not be completed because of connectivity problems, three were too busy to be interviewed. Fourteen participants completed interviews. Of the patients who refused/declined to participate in the trial, none were willing to be interviewed. We interviewed six recruiting doctors (face-to-face) and six on-site researchers (3 by phone calls and three face to face as they were on site, Supplemental table). Interviews lasted an average of 30 min (range, 15-55 min). Thematic analysis [38] was employed for the transcripts. We identified three key themes in this study (Fig. 5). Table 4 shows the detailed quotes from interviews.

**Table 4:** Description of the themes from the interview

Themes	Sub-themes	Example quotes
*Theme 1: Facilitators to the use of nutraceuticals for acute diarrhoea*	1.1 Effectiveness of nutraceuticals—expect doctor to prescribe ‘something’ to help with symptoms	“People come to see you and they would always expect you will provide “something” for them. Even if it is placebo, psychologically, they will feel better. It is hard in the Chinese context not to provide any medicines. The patient would feel you are not paying attention to them.” (52-year-old male doctor) “I come to hospital because there is nothing I can do by myself so I would expect the doctors to give me something to help with my symptoms” (45-year-old female patient) “It was very fast as I only took the medicines twice…then my diarrhoea stopped right away”. (37-year-old male patient) “could you please tell me what these medicines names are so I can buy (from pharmaceutical stores) by myself next time…..”. (31-year-old male patient) “These medicines are effective and most patients are happy with the treatment results”. (36-year-old male doctor) There were only two patients who expressed some concerns with side effects (39-year-old female): “I am bit worried as I do not know if there are any long term side effects although I asked the doctors and they said it is safe to take…. but I do not know which medicines I am taking….”. Two patients mentioned the one of the medicines was too big to swallow. (29-year-old female) “I found the yellow medicine was a bit too big to swallow.”
	1.2 Flexible schedule, friendly environment and easy to participate	“The schedule was very convenient for me as I have to work during the daytime so I can only come to follow up during evening…” (30-year-old female). “The students (research assistants) are very patient and always answer all the questions in detail, the process was simple and the diary was easy to fill..” (28-year-old male).
	1.3 Carefully designed, fully consented and novel trial	“To be honest, I never saw a trial like this with a really careful design following all standardised operating procedures….. especially, you have insurance for the trial…”. (50-year-old male doctor)
*Theme 2: Barriers to use nutraceuticals for acute diarrhoea*	2.1. Injections and antibiotics were commonly used for acute diarrhoea	“I used to use injections (injectable antibiotics) and it worked very well…” (49-year-old female patient) “if you do not prescribe it (antibiotics) to them, if they get angry, they might chase you with a knife….I do not want to face that….this is very common at the moment, it is hard to be a doctor in China….you might face a life threatening situation as you heard some doctors were killed by patients”(50-year-old male doctor). “usually we will give injectable antibiotics if patients insist on ‘injection’”. (36-year-old male doctor) “I do not know what medicines are inside the injection but I do feel the effect is fast with injections.” (36-year-old male patient) “In my past experiences, antibiotics are effective, especially for patients who used to use antibiotics, they won’t get better until you give them antibiotics as you do not know if they are viral or bacterial infections. Most of the tests are not accurate, so what you can do is to cover bacterial infections in case patients get worse…”. (52-year-old female doctor)
	2.2 Intensive doctor-patient relationship	“One argument was related to trial’s recruitment, another was during usual care patients to follow up. Although no one was injured, this indicates the extent of the distrust between doctors and patients. The argument related to trial’s recruitment, which resulted in police intervention occurred because a participant’s father was suspicious the trial’s therapy and came to the hospital to scold the recruiting doctor. They finally went to police office and checked all the documents. Of course they declined the trial after the argument. This is common in China. Although not so many patients are like this, you may occasionally face some patients who seem just to argue with doctors without any reasons.” (24-year-old male research assistant) “Currently, the health literacy among the Chinese public is low from what I heard and recently observed from this clinic. They do not have basic knowledge of health….. In China, some of doctors also do not have good knowledge of antibiotics, infection…..and they are used to using them (antibiotics)”. (24-year-old male research assistant) “it seems to me patients and doctors have had this habit (using injectable antibiotics for acute diarrhoea) for a long time. It is not easy to change a behaviour….. in a short time.” (24-year-old female research assistant)
	2.3 Nutraceutical therapy is not new to Chinese doctors	One doctor (50-year-old male) mentioned that he started to recruit patients with more prominent symptoms, which he was reluctant to do at the beginning: “I started to recruit patients with higher while blood cell, higher fever, severe belly cramping…”.
*Theme 3 Suggestions for future study*	3.1 Diary and stool samples	There were conflicting reports from patients regarding the use of the diary for symptom report. Nine interviewed patients found that the diary was easy to use. However, four mentioned they could not carefully read and fully understand the diary and one patient suggested some wording changes. “We observed that patients usually do not have stool when they came to hospital as they had diarrhoea symptom at home. In total, we only received a small number of (stool) samples and none were positive for bacterial pathogens. This might need to change in the future study…” (25-year-old male research assistant).
	3.2 Advocating this therapy for the public	“I found this is a very meaningful trial and I really hope we will know what those medications are so we can use them in the future or recommend them to families and friends”. (28-year-old male patient)
	3.3 Communication skills training	"don’t say the word ‘trial’ in Chinese as patients would feel you are treating them like laboratory animals. In reality, not all trials are like that and at least this trial is safe and it is phase 4. Instead saying something like ‘research’ would be better”. (42-year-old male doctor) “we received training from experienced peers who had been trained but we feel it is necessary to have more….”. (23-year-old male research assistant)
	3.4 Education and campaign for public	“If patients stop demanding antibiotics or requesting that antibiotics not be used, this will reverse the situation and consequentially influence doctors’ behaviour. It seems easier starting from patients/public rather than with doctors.” (42-year-old male doctor) “I usually follow a doctor’s advice as I do not know what will be good for me. If someone tells me that acute diarrhoea does not need antibiotics and there are additional side effects from an antibiotic, I won’t take it (antibiotic)…”. (30-year-old female patient)

*Factors facilitating* use of nutraceuticals to manage acute diarrhoea and recruitment included the expectation for doctors to prescribe “something” to relieve symptoms. Both doctors and patients had very positive views on the effectiveness of the nutraceuticals. All interviewed patients and doctors expressed satisfaction with their treatment effects. Some patients even asked what those medications were and where to purchase them. Most interviewed patients felt that this trial had a flexible follow-up schedule and that the research assistants and doctors were friendly and provided very detailed explanations. Most of the doctors and RAs considered this trial was carefully designed and the concept was new.

However, there were also *significant barriers* to use of nutraceuticals to manage acute diarrhoea and to recruitment. Most patients believed antibiotics were “effective” for acute diarrhoea and most doctors would use antibiotics if patients “demand” them, as they worried about patients’ satisfaction and subsequent confrontation. All doctors and most RAs mentioned the doctor-patient relationship was a big barrier for trust. There were two arguments during the recruitment period. Half of the doctors would consider antibiotics for acute diarrhoea even without patients’ demands as they believed antibiotics are effective and shorten patients’ symptom duration. Doctors reported that they were initially suspicious of the anticipated effect as berberine and loperamide were commonly used previously and they did not feel they worked, whilst curcumin was familiar to them but they had not used it. However, positive patient feedback increased their confidence in this trial. One doctor reported that he started to recruit more patients with higher symptom scores after he observed that patients could accept the therapy and it was seen to be effective. Only two patients complained that turmeric capsules were too big to swallow. As there had not been a randomised trial like this before [19], doctors agreed that it was hard to tell whether these medicines would really work until they saw the results.

There were *several suggestions* for improving the trial’s processes for future studies. Although nine interviewed patients found that the diary was easy to use, four mentioned they could not carefully read and fully understand the diary and one suggested some wording changes. The stool samples were challenging to collect as it was difficult for patients to produce a stool on demand. Communication skills training was suggested by several doctors for on-site research assistants to allow them to communicate effectively with patients. All research assistants and doctors commented on the need for public awareness campaigns to reduce the demand for antibiotics and relieve the pressure on doctors. Five patients recommended better provision of information of the adverse effects of unnecessary antibiotic use. Ten patients reported that they wanted the public to benefit as that they were surprised that they did not expect to recover so quickly without antibiotics. All participants expressed their willingness to recommend this therapy to others and felt this was a meaningful trial and would support using the trial medication.

## Discussion

### Summary of findings

Only 21% of patients screened for inclusion into this study were deemed to be eligible, and only half of those consented for study enrolment. The follow-up was excellent throughout; there was a very low rate of withdrawal and no safety concerns. Both doctors and patients were happy with the treatments and were willing to scale up or recommend to others. Most patients were happy with the diary and considered it easy to use, although some older patients found it more difficult. Blinding was successful.

The main barriers to recruitment are the strong belief that antibiotics are effective for the treatment of acute diarrhea and the often fraught doctor patient relationship in China. Doctors fear patient dissatisfaction and find it difficult to avoid the deeply entrenched routine of using injections and injectable antibiotics. During consultations many patients requested injections and antibiotics, increasing pressure on doctors to comply, and one patient complaint was only resolved by involving a local policeman.

Although this feasibility study was not powered to detect a difference between groups in the time to recovery, this study does provide support for a larger trial to document the effective use of alternatives to antibiotics for the treatment of acute diarrhoea in China. Although antibiotics are not recommended for diarrhea, they are commonly used in many places especially in LMICs [39–41]. Only 2% (3/136) of participants used antibiotics, in contrast to the 67% of the patients in routine care at the same hospital during the study period, when more than 60% of the prescribed antibiotics were given by injection, although the symptom severity is difficult to compare without a randomly assigned usual care control group. However, all usual care data came from patients diagnosed with acute diarrhoea without co-morbidity within the same age range during the same recruitment period. Up to 50% were eligible for inclusion but refused to participate in the trial.

### Comparison with the existing literature

There have been few well-designed studies on traditional Chinese medicines (TCM) as alternatives to antibiotics for relief of symptoms for acute diarrhoea [42, 43]. Many of those trials were with small sample size, majority of the trials were considered high risk of bias, mainly due to unclear concealment and no blinding [1]. Trial insurance requirements were only recently launched in China [44, 45] as one of the doctors mentioned that it was the first time he had seen a trial covered by insurance.

This feasibility study has provided evidence that it is possible to implement a trial like this in China in spite of the recruitment challenges observed. Other studies have also shown similar antibiotic use rates in China [46] despite the expansion of antibiotic stewardship in hospitals and the National Action Plan on containing antimicrobial resistance in 2016 [47]. One national survey on the antimicrobial stewardship programme (ASP) mentioned that although more than 65% of doctors were familiar with the ASP, only 46% of them had correct answers with ASP test [48]. This indicates the urgent need for further training for doctors.

### Strengths and Limitations

This is the first feasibility trial investigating the use of nutraceuticals to treat acute diarrhoea in China. A strength of this study is that we used both quantitative and qualitative methods to explore and more deeply understand the recruitment challenges. The interviews shed light on the underlying reasons for the observed high refusal rate and the suggestions from this interview study will be invaluable for future scaling up of studies in China. Although the study was under-powered to compare the effectiveness of each nutraceutical, each treatment resulted in symptoms resolving rapidly, and most patients and doctors felt the treatments were effective.

This study has limitations. First, most of the recruited patients experienced very mild symptoms [29]. We were unable to interview any of the patients who refused consent to participate. As discussed above, recruitment may have been influenced by the doctors’ complex relationship with patients in China [12]. Many of the recruited patients were young with a college education level, who might be expected to have lower expectations for antibiotics. We also did not systematically collect stool samples to determine whether bacterial, parasitic or viral infections were present; this was originally planned, but proved logistically difficult in the outpatient setting as patients were mostly unable to provide a stool specimen at the time of consultation.

### Implications for policy, practice and further research

Our results suggest that progression to a full randomised trial is feasible. Adequate onsite support will be needed as doctors are very busy and many lack research experience. Minor modifications will be needed to the diary to make it clearer and easier to complete though the completion rate was high (92%). The evidence that patients recovered within 48 h without antibiotics can be used to help doctors and patients accept using alternatives to antibiotics. Although no formal progression criteria were set at the start of this trial or written into the study protocol, the feasibility of a definitive trial was assessed against the objectives as set out in Table 5 alongside the data collected in the qualitative study.

**Table 5:** Feasibility objectives and endpoints of Diamond study

Feasibility objectives	Endpoints assessment
Eligibility: number of patients included and number excluded (+ reasons) from the trial.	The minimum representative sample size to be recruited from those eligible sufficient participants to allow the definitive trial results to be generalizable.
Recruitment: ability to recruit patients into the intervention from those attending primary care	The minimum recruitment rate per site per month to make the required sample size adequate for a definitive trial possible within a reasonable timeframe.
Retention: across the duration of the intervention and return of a fully completed diary	The minimum number of data completion in the diaries sufficient to allow the definitive trial results to be generalizable.
Trial medicine compliance	Do participants comply with intervention medicines sufficiently to make a definitive trial worthwhile?
Acceptability of the patient diaries, patients’ willingness to complete them, and the importance of telephone/text contact	Qualitative interview
Antibiotics and co-consultations are needed	The rates of antibiotics use and re-consultation
To inform sample size for future trials	The rate of outcome measures in the intervention groups compatible with conducting a definitive trial in China, with an achievable sample size, within a reasonable timeframe

From this study, we learnt that antibiotics were commonly used for acute diarrhoea in usual care in clinic (67%), which were often provided because of the doctors’ belief in the effect of antibiotics for acute diarrhoea and the long-established practice in using antibiotics, or the anxiety related to patient demands. This indicates that antibiotic stewardship programmes are urgently needed in many hospitals and clinics to address over-use of antibiotics especially for those with acute diarrhoea in China. Effective alternatives to antibiotics are likely to be an important intervention for these patients. A large fully powered trial is needed to define the effectiveness by comparing placebo, loperamide, berberine and loperamide + berberine in a wider range of patients with acute diarrhoea in China.

## Conclusion

Recruitment of patients with anything other than mild diarrhoea was very challenging in the current clinical environment in China. However, patients reported their symptoms recovered quickly. Patients need better information on the adverse effects of unnecessary antibiotic use. Use of loperamide and berberine may relieve symptoms as a viable alternative to antibiotics. This approach should be scaled up for further evaluation in a randomised controlled trial to investigate its effectiveness.

## Supplementary Information

**Additional file 1.**

**Additional file 2.**

**Additional file 3: Supplement Table.** Characteristics of interview participants

## Data Availability

The datasets used and/or analysed during the current study are available from the corresponding author on reasonable request.

## Abbreviations

DIAMOND: Diarrhoea Antibiotic Management using Over-the-counter Nutraceuticals in Daily practice
RCT: Randomised controlled trial
WHO: The World Health Organisation
TCM: Traditional Chinese medicines
OTC: Over-the-counter
