# Correction to: Diarrhoea Management using Over-thecounter Nutraceuticals in Daily practice (DIAMOND): a feasibility RCT on alternative therapy to reduce antibiotic use

**DOI:** 10.1186/s40814-021-00890-4

**Published:** 2021-08-18

**Authors:** Yanhong Jessika Hu, Xudong Zhou, Shanjuan Wang, Merlin Willcox, Colin Garner, David Brown, Taeko Becque, Beth Stuart, Zongru Han, Qin Chang, Michael Moore, Paul Little

**Affiliations:** 1 Murdoch Children’s Research Institute, Royal Children’s Hospital, Parkville, Victoria Australia; 2 Department of Paediatrics, The University of Melbourne, Parkville, Victoria Australia; 3 School of Public Health, Zhejiang University, 866 Yuhangtang Road, Zhejiang 310058, Hangzhou, China; 4 Affiliated Hospital of Jiading District Centre, Shanghai Institute of Health Science, No. 1 Chengbei Rd, Jiading Qu, Shanghai Shi, 201800 China; 5 Primary Care Research Centre, University of Southampton, Aldermoor Health Centre, Aldermoor Close, Southampton, SO16 5ST UK; 6 Antibiotic Research UK, Genesis 5, York Science Park, Heslington, YO10 5DQ York UK; 7 Alchemy Biomedical Consulting, St Johns Innovation Centre, Cowley Road, Cambridge, CB4 0WS UK

**Correction to: Pilot Feasibility Stud 7, 126 (2021)**

**https://doi.org/10.1186/s40814-021-00850-y**

Following publication of the original article [1], the authors reported an error in the Author contributions section. The abbreviated author’s name JYH should be updated to YJH.

The original article [1] has been updated.
