# Supplementary material for: Diarrhoea Management using Over-the-counter Nutraceuticals in Daily practice (DIAMOND): a feasibility RCT on alternative therapy to reduce antibiotic use
Source: Pilot Feasibility Stud. 2021 Jun 15;7:126. doi: 10.1186/s40814-021-00850-y (PMC8204461; doi:10.1186/s40814-021-00850-y)
Supplement: Supplementary file 1 — Additional file 1. [file 40814_2021_850_MOESM1_ESM.doc]

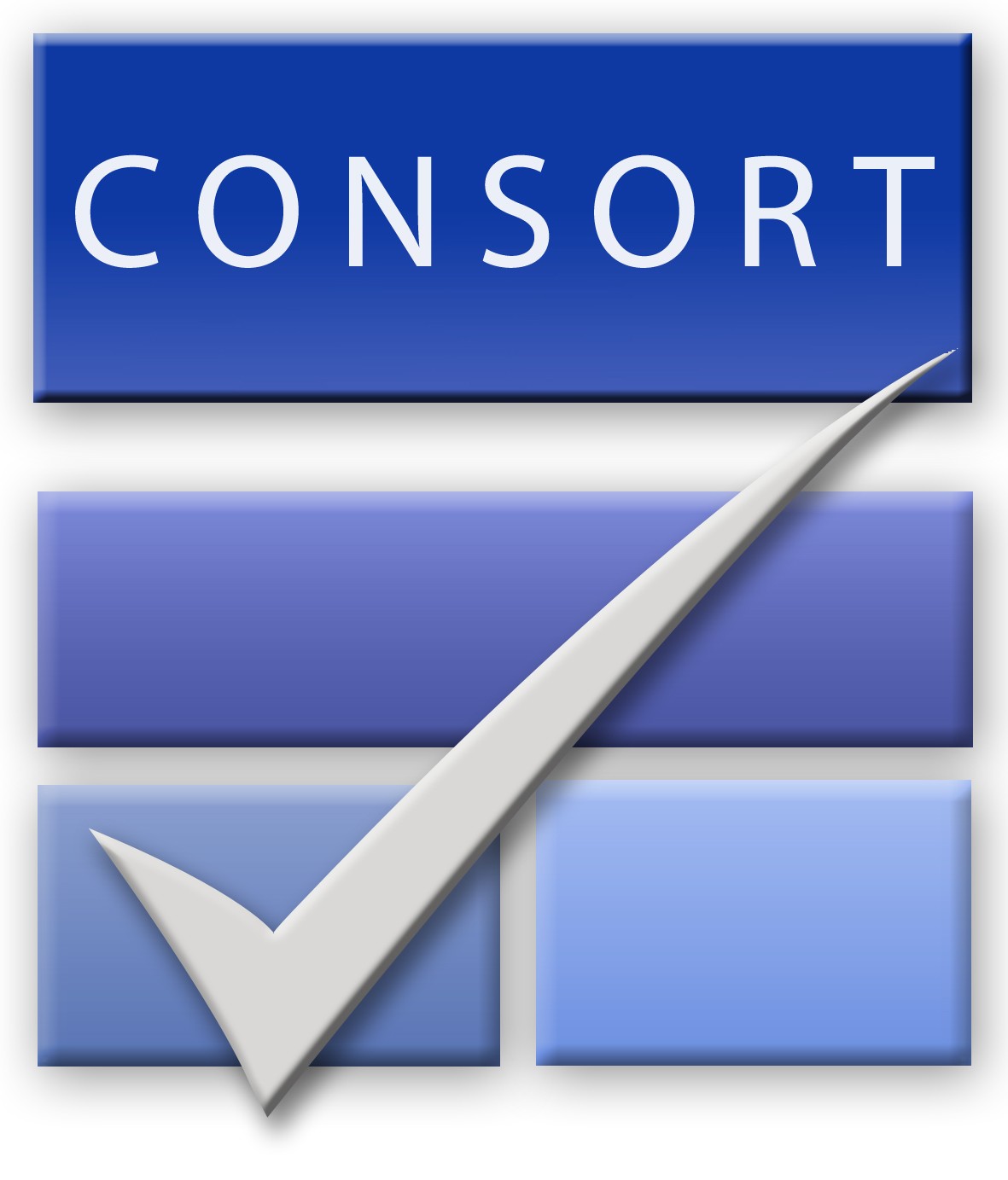
CONSORT 2010 checklist of information to include when reporting a pilot or feasibility trial*

| Section/Topic | Item No | Checklist item | Reported on page No |
| --- | --- | --- | --- |
| Title and abstract | | | |
|  | 1a | Identification as a pilot or feasibility randomised trial in the title | P1 Line 1-2 |
|  | 1b | Structured summary of pilot trial design, methods, results, and conclusions (for specific guidance see CONSORT abstract extension for pilot trials) | P1-2 Line 27-48 |
| Introduction | | | |
| Background and objectives | 2a | Scientific background and explanation of rationale for future definitive trial, and reasons for randomised pilot trial | P3-5 Line 76-133 |
|  | 2b | Specific objectives or research questions for pilot trial | P5 Line 123-133 |
| Methods | | | |
| Trial design | 3a | Description of pilot trial design (such as parallel, factorial) including allocation ratio | Page 9, |
|  | 3b | Important changes to methods after pilot trial commencement (such as eligibility criteria), with reasons | N/A |
| Participants | 4a | Eligibility criteria for participants | P5-6 Line 142-151 |
|  | 4b | Settings and locations where the data were collected | P5 Line 139-141 |
|  | 4c | How participants were identified and consented | P5 Line 142-144 |
| Interventions | 5 | The interventions for each group with sufficient details to allow replication, including how and when they were actually administered | P6 -7 Line 164-179 |
| Outcomes | 6a | Completely defined prespecified assessments or measurements to address each pilot trial objective specified in 2b, including how and when they were assessed | P5-6 Line 124-133 |
|  | 6b | Any changes to pilot trial assessments or measurements after the pilot trial commenced, with reasons | N/A |
|  | 6c | If applicable, prespecified criteria used to judge whether, or how, to proceed with future definitive trial | Table 5 |
| Sample size | 7a | Rationale for numbers in the pilot trial | Page 9 Line 229-237 |
|  | 7b | When applicable, explanation of any interim analyses and stopping guidelines | N/A |
| Randomisation: |  |  |  |
| Sequence  generation | 8a | Method used to generate the random allocation sequence | P6 Line 154-162 |
|  | 8b | Type of randomisation(s); details of any restriction (such as blocking and block size) | P6 Line 154-155 |
| Allocation  concealment  mechanism | 9 | Mechanism used to implement the random allocation sequence (such as sequentially numbered containers), describing any steps taken to conceal the sequence until interventions were assigned | P6 Line 156-159 |
| Implementation | 10 | Who generated the random allocation sequence, who enrolled participants, and who assigned participants to interventions | P6 Line 153-162 |
| Blinding | 11a | If done, who was blinded after assignment to interventions (for example, participants, care providers, those assessing outcomes) and how | P6 Line 161-162 |
|  | 11b | If relevant, description of the similarity of interventions | P6 Line 151-153 |
| Statistical methods | 12 | Methods used to address each pilot trial objective whether qualitative or quantitative | P7 Line 164-179 |
| Results | | | |
| Participant flow (a diagram is strongly recommended) | 13a | For each group, the numbers of participants who were approached and/or assessed for eligibility, randomly assigned, received intended treatment, and were assessed for each objective | P11 Line 267-273 |
|  | 13b | For each group, losses and exclusions after randomisation, together with reasons | P12 Line 284-298 |
| Recruitment | 14a | Dates defining the periods of recruitment and follow-up | P5 Line 139, page 8 Line 198 |
|  | 14b | Why the pilot trial ended or was stopped | N/A |
| Baseline data | 15 | A table showing baseline demographic and clinical characteristics for each group | P11-12 Line 281-292 |
| Numbers analysed | 16 | For each objective, number of participants (denominator) included in each analysis. If relevant, these numbers  should be by randomised group | Table 2 |
| Outcomes and estimation | 17 | For each objective, results including expressions of uncertainty (such as 95% confidence interval) for any  estimates. If relevant, these results should be by randomised group | Page 12-13, Line 308-332 |
| Ancillary analyses | 18 | Results of any other analyses performed that could be used to inform the future definitive trial | Page 15 Line 367-379 |
| Harms | 19 | All important harms or unintended effects in each group (for specific guidance see CONSORT for harms) | Page 13 318-322 |
|  | 19a | If relevant, other important unintended consequences | N/A |
| Discussion | | | |
| Limitations | 20 | Pilot trial limitations, addressing sources of potential bias and remaining uncertainty about feasibility | P18 Line 438-446 |
| Generalisability | 21 | Generalisability (applicability) of pilot trial methods and findings to future definitive trial and other studies | Page 18-19 Line 447-464 |
| Interpretation | 22 | Interpretation consistent with pilot trial objectives and findings, balancing potential benefits and harms, and  considering other relevant evidence | Page 16 Line 381-406 |
|  | 22a | Implications for progression from pilot to future definitive trial, including any proposed amendments |  |
| Other information | | |  |
| Registration | 23 | Registration number for pilot trial and name of trial registry | P1 Line 46 |
| Protocol | 24 | Where the pilot trial protocol can be accessed, if available | P1 Line 46 |
| Funding | 25 | Sources of funding and other support (such as supply of drugs), role of funders | P1 Line 47-49 |
|  | 26 | Ethical approval or approval by research review committee, confirmed with reference number | P20 Line 488 |

Citation: Eldridge SM, Chan CL, Campbell MJ, Bond CM, Hopewell S, Thabane L, et al. CONSORT 2010 statement: extension to randomised pilot and feasibility trials. BMJ. 2016;355.

*We strongly recommend reading this statement in conjunction with the CONSORT 2010, extension to randomised pilot and feasibility trials, Explanation and Elaboration for important clarifications on all the items. If relevant, we also recommend reading CONSORT extensions for cluster randomised trials, non-inferiority and equivalence trials, non-pharmacological treatments, herbal interventions, and pragmatic trials. Additional extensions are forthcoming: for those and for up to date references relevant to this checklist, see [www.consort-statement.org](http://www.consort-statement.org/).
