# Supplementary material for: Diarrhoea Management using Over-the-counter Nutraceuticals in Daily practice (DIAMOND): a feasibility RCT on alternative therapy to reduce antibiotic use
Source: Pilot Feasibility Stud. 2021 Jun 15;7:126. doi: 10.1186/s40814-021-00850-y (PMC8204461; doi:10.1186/s40814-021-00850-y)
Supplement: Supplementary file 2 — Additional file 2. [file 40814_2021_850_MOESM2_ESM.pdf]

DIAMOND Adverse Event/Serious Adverse Event reporting SOP

V 2

Role	Name	Signature	Date
SOP author	Jessika		
SOP review	Paul Little		
SOP review			

Effective Date		Review Date	

Revision History

Version	Revision	Date
1	New document.	30/03/ 2018
2		
3		

Definitions and abbreviations

Centre	The Zhejiang University department supporting recruitment to the trial.<br>In this trial the lead Centre is the University of Southampton and the specific department is Aldermoor Health Centre.<br>The coordinating Centres are the hospital site in Shanghai.

CCF	Coordinating Centre File, a file similar to the TMF held by the centres supporting the trial which holds all the information relevant to that centre.
CFDA	China Food and Drug Administration
CI	Chief Investigator, is in overall charge of the project.
CRF	Case Report Form, the form that collects all the data about participants.
CTA	Clinical Trials Authority
CTIMP	Controlled Trial of an Investigational Medicinal Product.
CTU	Clinical Trials Unit, a supporting unit often within a University.
DL	Development Lead (SOP); anyone with previous experience of the procedure / completing the procedure being described, who will take the lead in drafting the SOP or delegating specific section of the SOP to the appropriate person.
DG	Development Group (SOP); A group of approximately 2-4 personnel who are responsible for helping develop, maintain and improve the SOP system, consists of other suitably experienced members.
DM	Data Manager, an individual with responsibility for ensuring data is captured in an ethical manner and a useable format.
GCP	Good Clinical Practice, the regulations that govern the practice of researchers.
GMP	Good Manufacturing Practice, of IMP.
IMP	Investigational Medicinal Product
ISF	Investigator Site File, a file held by a Local Investigator containing all information they need to safely conduct the project.
LI	Local Investigator, the individual with responsibility for the conduct of the study at their site. In a CTIMP this has to be a medically qualified doctor or pharmacist.
PI	Principal Investigator, an Individual responsible for the safe and ethically conduct of the study, often leading a centre in academic research.
S(T)A	Study (Trial) Administrator a member of staff from the Centre.
S(T)C	Study (Trial) coordinator a senior member of staff who may have delegated tasks
S(T)M	Study (Trial Manager) a senior member of staff from the Centre who will have delegated tasks

	to run the project.
SOP	Standard Operating Procedure, specifies what should be done, when, where and by whom
Site	Primary care Centre that recruits into the study or trial
Sponsor	Zhejiang University
TMF	Trial Master File, a file containing all relevant information about the running of the project.

Contents

Revision History 1
Definitions and abbreviations 1
1. INTRODUCTION 3
2. DEFINITIONS 4
2.1 Adverse Reaction (AR) 4
2.2 Adverse Event (AE) 4
2.3 Serious Adverse Event (SAE) 4
2.4 CTIMP .... **Error! Bookmark not defined.**
2.5 MHRA 5
2.6 Main REC 5
2.7 Sponsor 5
2.7 Research Governance Office 5
2.8 Clinical Trials Unit 5
3. SCOPE 5
4. RESPONSIBILITIES 5
4.1 5
4.2 5
4.3 5
5. PROCEDURE 5
6. RELATED DOCUMENTS 7

1. INTRODUCTION

DIAMOND is an observational with a RCT study to exploring the alternative therapy to reduce antibiotic use in acute diarrhoea in adult in China. It is known that trials among adults suggest no benefit for using antibiotics for acute diarrhea except for travel diarrhea and confirmed bacteria

bacterial infections. Traditional Chinese medicine (TCM) are an acceptable and confirmed effect for relief symptom of diarrhea, however, we are aware of no randomized placebo-controlled trials available to support TCM as an alternative therapy in acute diarrhea therapy, therefore to reduce large amount of using antibiotics in acute diarrhea. A pilot of clinical trial in adults in China to ensure the therapy is effective and will be used in many other developing countries that they usually used antibiotics for acute diarrhea. It is difficult for clinicians to against the rising tide of antibiotic use just to tell patients to drink more water without any prescription. It might be good to have the alternative therapy to relieve symptoms. If any reduction in antibiotic prescribing is to be achieved, one of the key issue for patients and clinicians is the difficulty of knowing whether there are some medicines which can be used to show their care for patients in the local culture. It is also important to explore the effect on the acute diarrhea so we understand whether there is any side effect and other complications that we might need to understand in the future study, whether a large cluster scaling studies are necessary in the future.

The AE and SAEs we are interested in are the common known illness presentations such as severity of diarrhea and side effects of TCM e.g. constipation and rash.

2. DEFINITIONS

2.1 Adverse Reaction (AR)

In a CTIMP, any untoward and unintended response in a participant to an IMP which is related to any dose administered to that subject.

2.2 Adverse Event (AE)

Any untoward medical occurrence in a participant taking part in health care research that does not necessarily have a causal relationship with the research.

The following do not need to be reported as AEs if they are recorded as medical history/concomitant illness on the CRF at baseline.

Planned procedure, unless the condition for which the procedure was planned has worsened from the point of signing the consent form and appears to be related to the research.

Pre-existing conditions found as a result of screening procedures.

2.3 Serious Adverse Event (SAE)

Any adverse event/adverse reaction or unexpected adverse reaction that:

Results in death

Is life threatening

Requires in patient hospitalisation (defined as over 4 hours)

Results in persistent or significant disability/incapacity

Is a congenital anomaly or birth defect.

2.3 CFDA

China Food and Drug Administration. CFDA is the competent authority for China in relation to the clinical Trials Regulations. CFDA is the competent authority for the China in relation to the Medical Regulations 2016.

2.4 Main REC

The Research Ethics Committee undertaking the ethical review of the application.

2.5 Sponsor

The Zhejiang University as an institution takes responsibility for the initiation and management of this Research Study.

2.6 Research Governance Office

The office at the University of Southampton (UoS) tasked with ensuring that all Research Studies undertaken by members of UoS, or in its name, or sponsored by UoS are conducted in accordance with applicable legislation, guidelines and local policies.

2.7 Clinical Trials Unit

The Clinical Trials Committee will be responsible for the data management and ensuring quality through working with the Department of Biostatistics and Research Support.

3. SCOPE

This SOP is for use in the reporting of SAEs for the DIAMOND study and trial only. It is not intended to replace clinical care that is deemed appropriate for the individual.

4. RESPONSIBILITIES

4.1 Each PI at site is responsible for completing the DIAMOND Study SAE form when any SAE is found. This should be completed fully and returned by fax to the DIAMOND Study team on +86 57188208218 within 24 hours of becoming aware of the event. It is also possible, and preferred, to report the SAE using the Research Online system available at http://www.sda.gov.cn/.

4.2 The causality and expectedness will be re-assessed by the trial team of Professor Paul Little or Dr. Jessika Hu. This may be delegated to the local Co- Investigators in the supporting this study.

4.3 Also the guidelines for SAE and safety reporting to the regulatory authorities and Ethical Committees should be followed. To support this we will send out an overview of all SAEs that occurred every 3 months to study site. It will be the responsibility of the lead centre (University of Southampton) to inform the authorities and ethical committees involved. In general they like to receive a yearly update, but it could be there are local differences between the centres.

5. PROCEDURE

Legislation requires that SAEs be reported to the appropriate authorities in a timely manner. All Serious Adverse Events should be immediately reported to the following (timelines are given):

All SAEs occurring during the study should be reported on a specific form on the DIAMOND website within 24 hours. The SAE form is available for all patients included in the study as one of the surveys on the DIAMOND online system (named Research Online (RO)), and (depending on the organisation) also as a paper form.

The Recruiting clinician (RC) will fill in the DIAMOND SAE form as soon as he/she becomes aware a SAE occurred. This form will be faxed to the coordinating centre as soon as possible, who will immediately fill in the SAE form of the patient involved on the DIAMOND website (http://www.chictr.org.cn/index.aspx) . To make sure the Study manger is aware that a SAE occurred and that the related data is entered on the website, the RC should inform the Study team by telephone as well. Alternatively the RC can fill in the SAE form on the website directly. SAEs that occur during the study must also be documented in the subject's medical record. On completion of an SAE on the DIAMOND website, an automatic email is generated to alert the trial management team. The Chief or Principal Investigators, on behalf of the sponsor, will assess whether the SAE is a SUSAR (Suspected Unexpected Serious Adverse Reaction).

A SUSAR is any adverse reaction that is classed as serious and is suspected to be caused by the investigational medicinal product (IMP) and is not consistent with the information about the (IMP) in the Summary of Product Characteristics (SmPC). The IMP used for this study is the well-known antibiotic Cipro, it is being used within its licence therefore SUSARs are not expected.

If the CI/PI (on behalf of the sponsor) decides the SAE should be classified as a SUSAR, all medically qualified co-Investigators will be informed immediately and delegated, by the sponsor, to report the SUSAR to their competent authority (CA) and ethics committee (EC). SUSARs should only be entered into EudraVigilance CT. For a SUSAR which is fatal or life threatening, the Chief Investigator, on behalf of the sponsor, has 7 days after the reporting RC becoming aware of the event to report the SUSAR to the CA and to the EC. For a SUSAR which is not fatal or life threatening, the Chief Investigator, on behalf of the sponsor, has 15 days to report this event to the CA and to the EC. The SUSAR is recorded in the participants' medical notes and the participant is followed up. All SUSAR's should be included in the annual safety report.

**For how to fill in a SAE form on the website see the Manual for the DIAMOND Online System **

1- days. However, in the case of a 3rd SAE the sponsor should be informed immediately by faxing a completed DIAMOND SAE report to the following number: +86571-88208221.

It could be that during assessment of the reported SAEs, additional information is requested. If so, it should be provided in a timely fashion to ensure accurate follow-up of each case.

The Table 1Timelines and responsibilities in SAEs page 8 describes the timelines and responsibilities.

6. RELATED DOCUMENTS

The DIAMOND Signature and delegation log will support the completion of AE/SAE reporting.

The DIAMOND IMP handling document describes the handling of the IMP, the training and delegation logs indicate the site personnel who are deemed responsible for completing the SAE reporting mechanism. The DIAMOND IMP handling SOP describes the procedures for IMP handling through the whole trial processes.

The DIAMOND Unblinding SOP describes the processes required for unblinding a participant.

Table 1Timelines and responsibilities in SAEs

What	Who	When	How	To whom
SAE	CI or Sponsor	Within 24 hours of the staff becoming aware of the event	SAE report form for project	Main REC
Urgent safety measures	CI or Sponsor	immediately	By telephone or by notice in writing setting out the reasons and the plan for further action	Main REC, local REC if by PI
SUSARs	CI or Sponsor	Within 7 days	In writing	CFDA
Progress reports	Sponsor or Sponsor's legal representative, always signed by CI	Annually (starting 12/12 after the favourable opinion)	From HRA website	Main REC
Declaration of the conclusion or early termination of the research	CI or Sponsor	Within 90 days (conclusion) within 15 days (early termination)<br>NB the end should be defined in the Protocol	From HRA website	Main REC
Summary of final report	CI or Sponsor	Within one year of the conclusion of the research	No standard format. The summary should include information on whether the project achieved its objectives, the main findings and arrangements for publication and dissemination to participants.	Main REC
