# Supplementary material for: Diarrhoea Management using Over-the-counter Nutraceuticals in Daily practice (DIAMOND): a feasibility RCT on alternative therapy to reduce antibiotic use
Source: Pilot Feasibility Stud. 2021 Jun 15;7:126. doi: 10.1186/s40814-021-00850-y (PMC8204461; doi:10.1186/s40814-021-00850-y)
Supplement: Supplementary file 3 — Additional file 3: Supplement Table. Characteristics of interview participants [file 40814_2021_850_MOESM3_ESM.docx]

Additional file 3 Supplement table --Characteristics of interview participants

| **Interviewee** | **No** | **Gender** | **Age** | **Education**  **background** | **Working experiences**  **( Years)** |
| --- | --- | --- | --- | --- | --- |
| **Doctors** | 1 | Male | 42 | Bachelor | 13 |
|  | 2 | Male | 36 | Bachelor | 11 |
|  | 3 | Male | 42 | Master degree | 12 |
|  | 4 | Female | 52 | Bachelor | 28 |
|  | 5 | Male | 50 | Bachelor | 25 |
|  |  |  |  |  | **School year** |
| **Research Assistants (RAs)** | 1 | Male | 24 | Master | Year 3 |
|  | 2 | Female | 22 | PhD | Year 2 |
|  | 3 | Female | 24 | Master | Year3 |
|  | 4 | Female | 25 | PhD | Year1 |
|  | 5 | Male | 23 | Master | Year 3 |
|  | 6 | Female | 23 | Master | Year1 |
|  |  |  |  | **Hometown** | **Intervention groups** |
| **Patients** | 1 | Male | 23 | Shanghai | Loperamide & berberine |
|  | 2 | Male | 37 | Shanghai | Loperamide & berberine |
|  | 3 | Female | 39 | Anhui | Loperamide & turmeric & berberine |
|  | 4 | Female | 29 | Shanghai | Loperamide & turmeric |
|  | 5 | Female | 49 | Shanghai | Loperamide & turmeric & berberine |
|  | 6 | Female | 30 | Jiangxi | Loperamide & turmeric & berberine |
|  | 7 | Male | 40 | Shandong | Loperamide only |
|  | 8 | Male | 22 | Heilongjiang | Loperamide only |
|  | 9 | Female | 45 | Shanghai | Loperamide & turmeric |
|  | 10 | Female | 34 | Shanghai | Loperamide & berberine |
|  | 11 | Female | 32 | Shanghai | Loperamide & turmeric |
|  | 12 | Male | 28 | Fujian | Loperamide & turmeric & berberine |
|  | 13 | Male | 31 | Shanghai | Loperamide only |
|  | 14 | Male | 36 | Hunan | Loperamide & berberine |
